# Next Generation Sequencing for Detecting Somatic *FAS* Mutations in Patients With Autoimmune Lymphoproliferative Syndrome

**DOI:** 10.3389/fimmu.2021.656356

**Published:** 2021-04-29

**Authors:** Marta López-Nevado, Jorge Docampo-Cordeiro, José T. Ramos, Rebeca Rodríguez-Pena, Celia Gil-López, Silvia Sánchez-Ramón, Juana Gil-Herrera, María J. Díaz-Madroñero, María A. Delgado-Martín, Pablo Morales-Pérez, Estela Paz-Artal, Aude Magerus, Frederic Rieux-Laucat, Luis M. Allende

**Affiliations:** ^1^ Immunology Department, University Hospital 12 de Octubre, Madrid, Spain; ^2^ Instituto de Investigación Sanitaria Hospital 12 de Octubre (imas12), Madrid, Spain; ^3^ Pediatrics Department, University Hospital Clínico San Carlos, Madrid, Spain; ^4^ Immunology Department, University Hospital La Paz, Madrid, Spain; ^5^ Immunology Department, University Hospital Clínico San Carlos, Madrid, Spain; ^6^ Immunology Department, University Hospital Gregorio Marañón, Madrid, Spain; ^7^ School of Medicine, University Hospital 12 de Octubre, Complutense University of Madrid, Madrid, Spain; ^8^ Université de Paris, Laboratory of Immunogenetics of Pediatric Autoimmune Diseases, Imagine Institute, INSERM UMR 1163, Paris, France

**Keywords:** ALPS, ALPSsFAS, autoimmunity, lymphoproliferation, malignancy, somatic, NGS, ALPS-like

## Abstract

Autoimmune lymphoproliferative syndrome (ALPS) is a primary immune regulatory disorder clinically defined by chronic and benign lymphoproliferation, autoimmunity and an increased risk of lymphoma due to a genetic defect in the FAS-FASL apoptotic pathway. Genetic defects associated with ALPS are germinal and somatic mutations in *FAS* gene, in addition to germinal mutations in *FASLG, FADD, CASP8* and *CASP10* genes. The accumulation of CD3+TCRαβ+CD4-CD8- double negative T-cells (DNT) is a hallmark of the disease and 20-25% of ALPS patients show heterozygous somatic mutations restricted to DNT in the *FAS* gene (ALPS-sFAS patients). Nowadays, somatic mutations in the *FAS* gene are detected through Sanger sequencing in isolated DNT. In this study, we report an ALPS-sFAS patient fulfilling clinical and laboratory ALPS criteria, who was diagnosed through NGS with a targeted gene panel using DNA from whole blood. Data analysis was carried out with Torrent Suite Software and variant detection was performed by both germinal and somatic variant caller plugin. The somatic variant caller correctly detected other six ALPS-sFAS patients previously diagnosed in the authors’ laboratories. In summary, this approach allows the detection of both germline and somatic mutations related to ALPS by NGS, avoiding the isolation of DNT as the first step. The reads of the somatic variants could be detected even in patients with DNT in the cut off limit. Thus, custom-designed NGS panel testing may be a faster and more reliable method for the diagnosis of new ALPS patients, including those with somatic *FAS* mutations (ALPS-sFAS).

## Introduction

Autoimmune lymphoproliferative syndrome (ALPS), initially termed Canale-Smith syndrome (1), is a rare Primary Immune Regulatory Disorder (PIRD) defined by childhood onset chronic and benign lymphoproliferation, autoimmune phenomena mainly in form of multilineage cytopenia and an increased risk of lymphoma due to an impairment of lymphocyte homeostasis (2–4). The accumulation of polyclonal CD3+TCRαβ+CD4-CD8- double negative T-cells (DNT), elevated serum levels of interleukin-10 (IL-10), soluble FAS ligand (sFASL) and vitamin B12 are considered biomarkers of ALPS (5). Lymphocyte homeostasis is affected by defective FAS-mediated apoptosis and in most ALPS patients the apoptotic defect is consequence of inherited (germline) or acquired (somatic) mutations in the *FAS* gene (ALPS-FAS and ALPS-sFAS patients, respectively). Less frequently, germinal mutations in the *FASLG* (ALPS-FASLG), *FADD* (ALPS-FADD)*, CASP10* (ALPS-CASP10) and *CASP8* (ALPS-CASP8) genes can occur (2, 3, 5–10). Although ALPS is a genetically well-defined syndrome, in approximately 20% of ALPS-suspected cases no mutations have yet been identified and they are classified as ALPS-unknown (ALPS-U) (5). Combination of somatic and germinal *FAS* mutations, involvement of other ALPS-phenotype-modifiers genes such as *UNC13D*, *XIAP, PRF1*, *CASP10*, as well as numerous genetic defects outside the FAS-FASL pathway leading to an ALPS-like phenotype have been described (10–16).

Heterozygous acquired or somatic mutations in the *FAS* gene are found in 20-25% of ALPS patients (10, 13, 17). These mutations are mainly restricted to the exons 7, 8 and 9 of *FAS* gene, mostly detectable in DNT-cell population, but also in approximately 10-20% of single-positive mononuclear cells (18). Phenotypically, DNT derives from both CD4 and CD8 effector memory T-cells with CD45RA expression, representing a subset of terminally differentiated T-cells that should have been deleted by apoptosis (19). Although elevated DNT is a required criterion of the disease, its pathologic role in ALPS remains unclear, but as ALPS-FAS and ALPS-sFAS are clinically indistinguishable, these cells could contribute to disease pathogenesis (10, 13).

Decisional trees for the diagnosis of ALPS have been proposed (5, 14, 20). It is established that if the clinical features of ALPS are present, DNT immunophenotyping and ALPS-biomarkers assessment are the first step of the algorithm (14). If DNT and any other ALPS-biomarkers are elevated, ALPS suspicion is confirmed and sequencing of the ALPS-related genes is mandatory. If no germinal mutation is found in these genes, searching for a somatic *FAS* mutation should be performed through Sanger sequencing in isolated DNT. Sanger sequencing can be a useful and fast tool for sequencing of specific genomic regions. However, Next Generation Sequencing (NGS) technologies such as targeted gene panels and whole exome sequencing have become powerful tools that allow simultaneous screening of heterogeneous genetic diseases as ALPS and ALPS-like disorders (14).

Generally, DNT represent less than 3% of total leukocytes (17). The finding of somatic mutations in the *FAS* gene requires the isolation of DNT population, a costly and time-consuming technique that may not be feasible in some routine laboratories. Based on the classical NGS application for detecting a relatively low mutational burden (21–23), we propose the possibility of detecting somatic mutations by DNA sequencing from whole blood using a targeted gene panel. This approach could allow the simultaneous screening of ALPS patients with both germline mutations in the ALPS related genes and somatic mutations in the *FAS* gene.

In this study we report a new case of ALPS-sFAS diagnosed by NGS through a targeted gene panel. The application of the somatic variant caller was validated with the detection of somatic mutations in other ALPS-sFAS patients previously diagnosed in the authors’ laboratories (13, 24, 25).

## Methods

### DNT Immunophenotyping

For direct immunofluorescence, peripheral whole blood was incubated using the corresponding monoclonal antibodies as previously described (14). Cellular acquisition was performed using a Beckman Coulter Navios cytometer (Beckman Coulter, Miami, FL, USA) and data were analyzed with Kaluza 2.1 software (Beckman Coulter).

Eight ALPS-sFAS patients were included in this study showing different DNT percentages (from 4 to 30.7%) (**Table 1**).

**Table 1:** ALPS parameters.

	P1	P2	P3	P4	P5	P6	P7	P8	Reference values
**Age (years)**	1	56	47	56	30	26	23	12	–
**CD3+ TCRαβ+ CD4-CD8- (DNT) (%)**	5.2	30.7	6.1	5.5	18	12	27	4	0-4
**Serum biomarkers(pg/mL)**									
**VitB12**	1911	>2000	>2000	>2000	1697	ND	ND	2098	197-771
**sFASL**	>1000	>1000	682	>1000	>1000	>1000	>1000	800	0-250
**IL-10**	460	98	66	128	32	62	110	24	0-20
**Serum Immunoglobulins (mg/dL)**									
** IgG**	1510	975	1410	1140	901	2385	ND	1230	600-1230
** IgA**	255	328	595	454	35	596	ND	300	30-200
** IgM**	131	45	35	94	<5	72	ND	174	50-200

ND, not determined.

### ALPS Biomarkers

Serum levels of IL-10 (Bender MedSystems, Labclinics, Madrid, Spain) and sFASL (R&D, Vitro, Madrid, Spain) were measured in duplicate by enzyme-linked immunosorbent assay. Vitamin B12 were measured by electro-chemiluminescence immunoassay (Beckman Coulter). Serum immunoglobulin concentrations were determined by nephelometry (Beckman Coulter) (14).

### Isolation of DNT

DNT were isolated by immunomagnetic cell separation (MACS) using a double-negative T-cell isolation Kit (Miltenyi Biotec GmbH, Germany) according to the manufacturer’s instructions as previously described (14).

### FAS-Induced Apoptosis

Functional studies for FAS-mediated apoptosis was performed as previously described (14) for screening of ALPS patients.

### Targeted Next Generation Sequencing Gene Panel

Genomic DNA was extracted from EDTA blood samples and isolated DNT-cells using a MagNa Pure Compact Nucleic Acid Isolation Kit (Roche, Basel, Switzerland).

NGS was run in an Ion Torrent PGM platform (Thermo Fisher Scientific, Waltham, Massachusetts, USA) using a targeted gene sequencing with an inhouse designed panel of 192 genes involved in primary immunodeficiencies (Ampliseq, Thermo Fisher Scientific) including all the ALPS-related genes.

Ion Torrent adapter-barcode ligated libraries were generated with 20 ng of DNA using the Ion AmpliSeq Library Kit 2.0 and Ion Xpress Barcode Adapter 1-16 Kit according to the manufacturer’s procedures, obtaining 300-bp fragments of PCR. Emulsion PCR was done on the Ion OneTouch 2 Instrument with Ion PGM Template OT2 200 Kit and finally the amplicons were sequenced using Ion PGM Sequencing Kit and Ion 318 Chip Kit v2 according to the manufacturer’s procedures (all reagents from Thermo Fisher Scientific). Coverage analysis was checked in Torrent Suite (Thermo Fisher Scientific) through the plugin Coverage Analysis.

### Variant Caller, Annotation and *In Silico* Prediction

Data analysis was carried out with Torrent Suite Software (version 5.12). Data produced were aligned and mapped to the human GRCh37 (hg19) genome assembly by Torrent Mapping Alignment Program (TMAP). For germline variants, the variant caller plugin with default setting of “Generic PGM Germ Line Low Stringency” (Thermo Fisher Scientific) was performed.

Somatic variants were detected by customization of the default setting of somatic variant caller plugin from Torrent Suite Software (TVC, v5.12. Thermo Fisher Scientific) considering only the exons 7, 8 and 9 of *FAS* gene. The following parameters were adapted from the somatic variant caller of Torrent Suite server: minimum allele frequency permitted was 0.01, minimum allele coverage was defined as 3 reads, no minimum coverage on either strand was defined but those variants with strand bias above 0.85 and strand bias *pvalue* below 0.05 was filtered out.

Variant annotation was performed with Ion Reporter Software (version 5.10). The software Integrative Genomics Viewer (IGV) (26) was used to inspect the aligned reads (bam files) of each sample in order to check the consistency of nucleotide calls. VarSome (27) was used for *in silico* pathogenicity prediction and for a comprehensive interpretation of the variants.

The somatic *FAS* mutations were detected with this approach in seven out eight patients. It is important to consider that in the patient 8 (P8) which we could not be able to detect the somatic mutation, he had the DNT in the limit for our consideration as an ALPS patient (required criterium). In P1 and P4, the mutations were detected even with slightly high DNT.

### Sanger Sequencing

Germline and somatic variants were confirmed by Sanger sequencing using the appropriate specific primers.

### Statistics

Sensitivity and specificity of the assay was calculated considering sensitivity as true positive rate (87.5% of ALPS-sFAS patients were correctly identified) and specificity as true negative rate (93.1% of controls were correctly identified).

## Results

### Patients

Patient 1 (P1) was admitted to the hospital with fever and exanthema at ten months of life. One year later he suffered from another febrile episode with exanthema and ALPS-suspected phenotype: splenomegaly, lymphadenopathies and bicytopenia (thrombocytopenia and neutropenia) and positive anti-smooth muscle antibodies. DNT, vitamin B12, IL-10, sFASL and immunoglobulins G and A were elevated (**Table 1**) confirming the ALPS suspicion. Patients 2-8 (P2-P8) are ALPS-sFAS patients previously diagnosed in the authors’ laboratories (**Table 1** **)** (13, 24, 25). The studies involving human participants were reviewed and approved by Comité Ético de Investigación Clínica, University Hospital 12 de Octubre and by ethic committee of CPP Ile-de-France (CPP IDF2 DC-2014-22722015-03-03 AF).

### Identification of Germinal and Somatic Mutations Through Targeted Next Generation Sequencing

**Figure 1:**
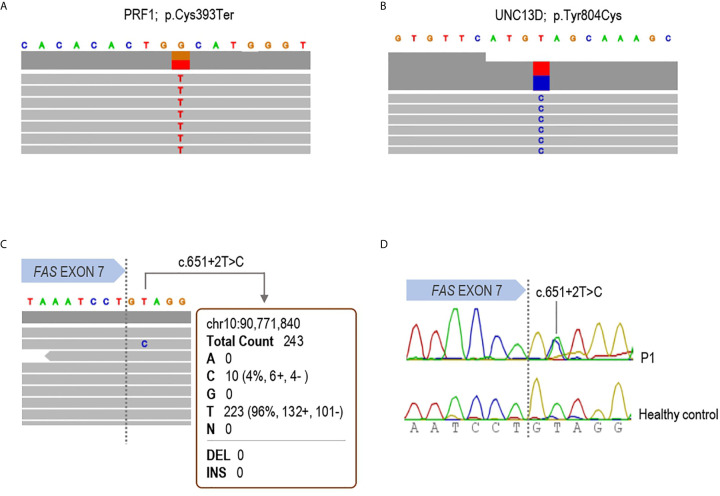
Molecular diagnosis of patient 1. IGV browser visualization of the targeted Next Generation Sequencing results in patient 1 is shown. **(A)** Germline heterozygous p.Cys393Ter variant in the *PRF1* gene; **(B)** Germline heterozygous p.Tyr804Cys substitution in the *UNC13D* gene and **(C)** the somatic mutation c.651+2T>C detected in whole blood DNA in the *FAS* gene. The variant was read 10 times with a variant allele frequency of 4.2%. **(D)** Sanger sequencing in DNA from isolated DNT confirming the somatic mutation in the patient.

NGS using DNA extracted from whole blood of P1 (**Figure 1**) revealed two germline heterozygous mutations in *PRF1* (c.1179C>A; p.Cys393Ter) and *UNC13D* (c.2411A>G; p.Tyr804Cys) genes (**Figures 1A, B**), both considered as ALPS-phenotype-modifiers but not ALPS-causing genes (11, 12, 15). Functionally, intracellular perforin staining and degranulation assay in NK cells were similar to the healthy control tested (data not shown). No germline variant was found in the ALPS-related genes. Given the great suspicion of ALPS and since somatic mutations in *FAS* gene are the second most frequent cause of ALPS, a customized somatic variant calling considering only the exons 7, 8 and 9 of *FAS* gene was carried out. A splicing variant in intron 7 (c.651+2T>C) previously reported as pathogenic in germinal line was detected in ALPS-FAS patients [referred as p.Pro201fs204Ter in (10, 28)]. The Variant Allele Frequency (VAF) got in the experiment was 4.2%. It was read 10 times out of a total of 243 reads (**Figure 1C**). For somatic variant confirmation, DNT from P1 were isolated through MACS separation, DNA was extracted and somatic variant in intron 7 of *FAS* gene was detected by Sanger sequencing (**Figure 1D**).

### Customized Somatic Variant Caller Validation

After detecting the somatic variant in P1 (c.651+2T>C) by NGS using DNA from whole blood, it was necessary to validate the somatic variant caller analyzing samples from seven other ALPS-sFAS patients previously diagnosed in the authors’ laboratories by Sanger sequencing in isolated DNT (P2-P8) (13, 24, 25).

**Figure 2:**
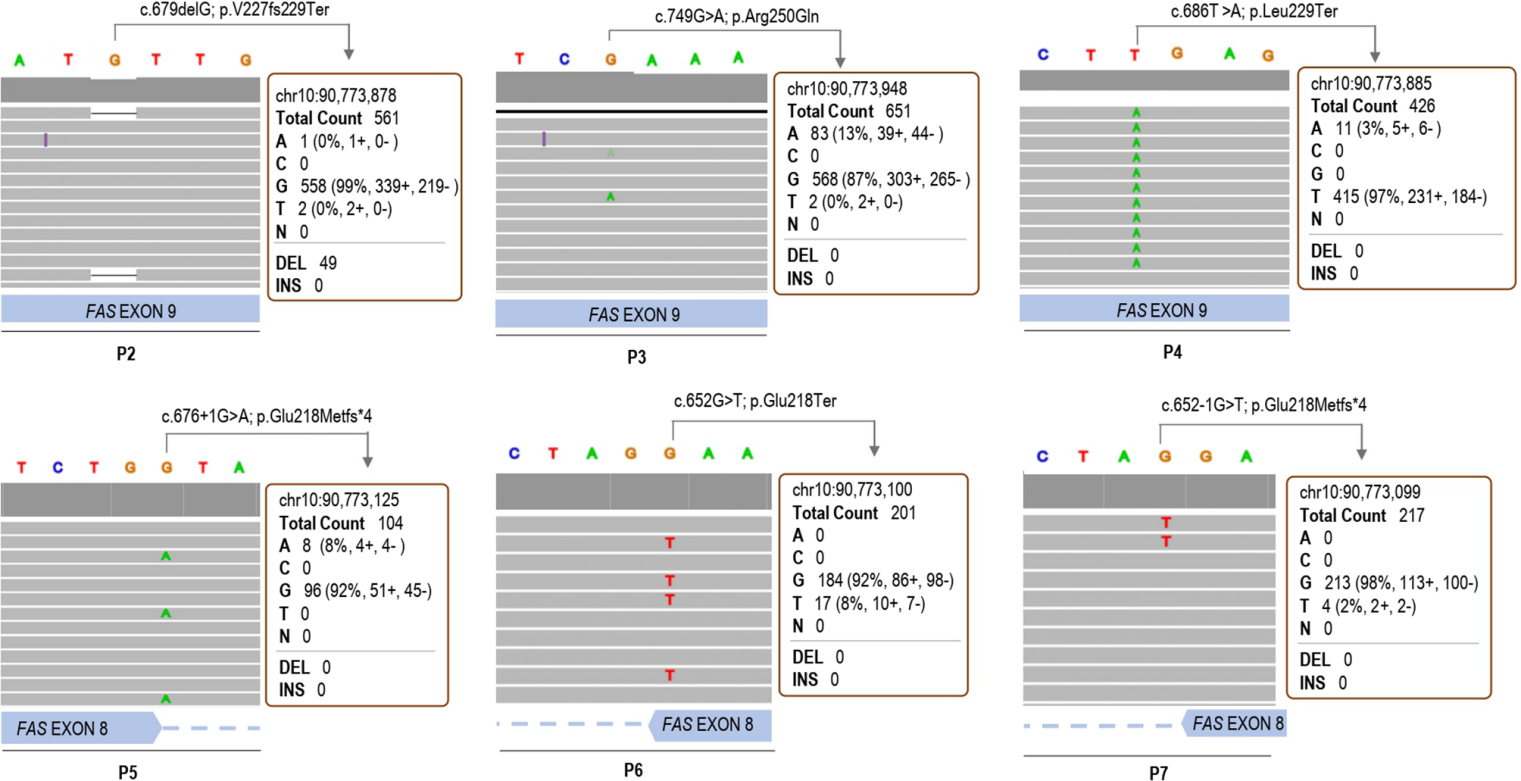
IGV browser visualization of the somatic variant calls through the customized variant caller plugin in patients 2, 3, 4, 5, 6 and 7.

To ascertain the quality of the somatic variants identified by our method, forty-four samples from non-ALPS patients were also included (C1-C44). In total, nine different variants were called in the 52 samples analyzed (**Table 2**). Strand bias, as the significantly different genotype inferred from the positive and negative strand, was around 0.5 in all cases, ensuring a good reads balance in both strands thus ruling out possible NGS errors. Somatic mutations of P1, P2, P3, P4, P5, P6 and P7 were correctly detected with a VAF of 4.2%, 7.6%, 11.5%, 2.7%, 7.9%, 8.7% and 1.9%, respectively **(** **Table 2** **)**. P8, with three base-pair deletion (c.812_814del; p.Ala271del) (25) was not detected by the somatic variant caller plugin, although the deletion was observed once in the IGV browser. Three out of five remaining variants (C18, C32, RV) had frequencies around 50% suggesting being germline heterozygous variants and the other two (P1, P4, C5) were somatic variants with frequencies of 2.1% and 2.9% (**Table 2**). All somatic mutations and variants calls were visually checked with the IGV **(** **Figure 2** **)**. Based on the experimental data, the minimum depth of coverage of 101 reads allows to detect a VAF of 7.9% (case of P5). When the depth reaches 212 reads, we can detect a minimum VAF of 1.9% (case of P7).

**Table 2:** Somatic variant caller report.

Sample	Chrom:position	Ref	Var	VAF	Cov	Allele cov	Allele cov -	Allele cov +	Strand Bias
**P1**	chr10:90771840	T	C	4.2	239	10	6	4	0.5318
**P2**	chr10:90773878	G	–	7.6	394	30	20	10	0.5125
**P3**	chr10:90773947	G	A	11.5	399	46	21	25	0.5497
**P4**	chr10:90773876	T	A	2.7	367	10	5	5	0.6237
**P5**	chr10:90773125	G	A	7.9	101	8	4	4	0.5148
**P6**	chr10:90773100	G	T	8.7	195	17	10	7	0.6249
**P7**	chr10:90773099	G	T	1.9	212	4	2	2	0.5279
**P1,P4**	chr10:90771774	A	G	2.1	141	3	2	1	0.6497
**C5**	chr10:90773123	T	C	2.9	104	3	2	1	0.5451
C18*	chr10:90771767	G	A	54.2	172	96	56	40	0.5219
C32*	chr10:90774155	C	T	48.1	395	190	98	92	0.5337
RV*	chr10:90771829	T	C	54.1	206	112	51	61	0.5133

Ref, genome reference nucleotide; Var, genome reference substitution; VAF, variant allele frequency; Cov, total coverage in each position; Allele cov, variant reads number; Allele cov -, variant reads in reverse strand; Allele cov, variant reads in forward strand; P, patient; C, control; RV, Recurrent variant presented in fourty-six samples. *Germline heterozygous variants.

Variant annotation and pathologic interpretations for new variants were performed with Ion Reporter Software and VarSome, respectively **(** **Table 3** **)**. Somatic mutations previously described in P2-P7 (13, 24, 25) and germline variants were excluded from variant annotation and *in silico* analyses. Somatic variant of P1 (c.651+2T>C) was predicted as pathogenic and was previously reported as disease causing in ALPS-FAS patients (10, 28). The somatic variant detected in both P1 and P4 (c. 587A>G) was not found in Clinvar and was classified as benign (twenty-one benign versus no pathogenic scores), but it was not detected in Sanger sequencing from their isolated DNT (data not shown). The somatic variant found in C5 (c.675T>C) is probably a benign synonymous variant that not predicted an aberrant splicing (27, 29) (**Table 3**). Although we cannot exclude that these two somatic variants are present in another cell population in the three cases (c. 587A>G in P1 and P4 and c.675T>C in C5), they have been considered as false positive variants. Data show that sensitivity of the assay is 87.5% and the specificity is 93.1%. These results are obtained without annotation and interpretation of the variants. If we consider the no pathogenic predictors’ interpretation for the two somatic variants observed in 3 samples, the specificity would be considered 100%.

**Table 3:** Variant annotation of unreported somatic variants.

Sample	Chrom:position	Ref	Var	Type of variant	Var effect	Coding	Protein	gnomAD (He)	Predictors of Pathogenicity (damaging/benign) *
**P1,P4**	chr10:90771774	A	G	Somatic	Missense	c.587A>G	p.Gln196Arg	NR	0/21
**C5**	chr10:90773123	T	C	Somatic	Synonymous	c.675T>C	p.(=)	NR	0/1

Ref, genome reference nucleotide; Var, genome reference substitution; gnomAD, allele frequency in the gnomAD browser; He, number of heterozygotes reported in gnomAD browser; NR, Not reported.

*Predictors of pathogenicity available from VarSome. The number of damaging versus benign scores are shown.

### FAS-Mediated Apoptosis

Nowadays, FAS-mediated apoptosis is not considered essential for the diagnosis of ALPS (accessory criterion) (5), as some ALPS patients (ALPS-sFAS or ALPS-FASLG) reported normal values of FAS-mediated apoptosis (14). In the case of ALPS-sFAS patients a small percentage of PBMCs (DNT cells) carry the *FAS* mutation, while in the case of ALPS-FASLG, FAS-mediated apoptosis is overlooked, therefore it’s necessary to demonstrate an impaired activation-induced cell death (AICD) (14).

**Figure 3:**
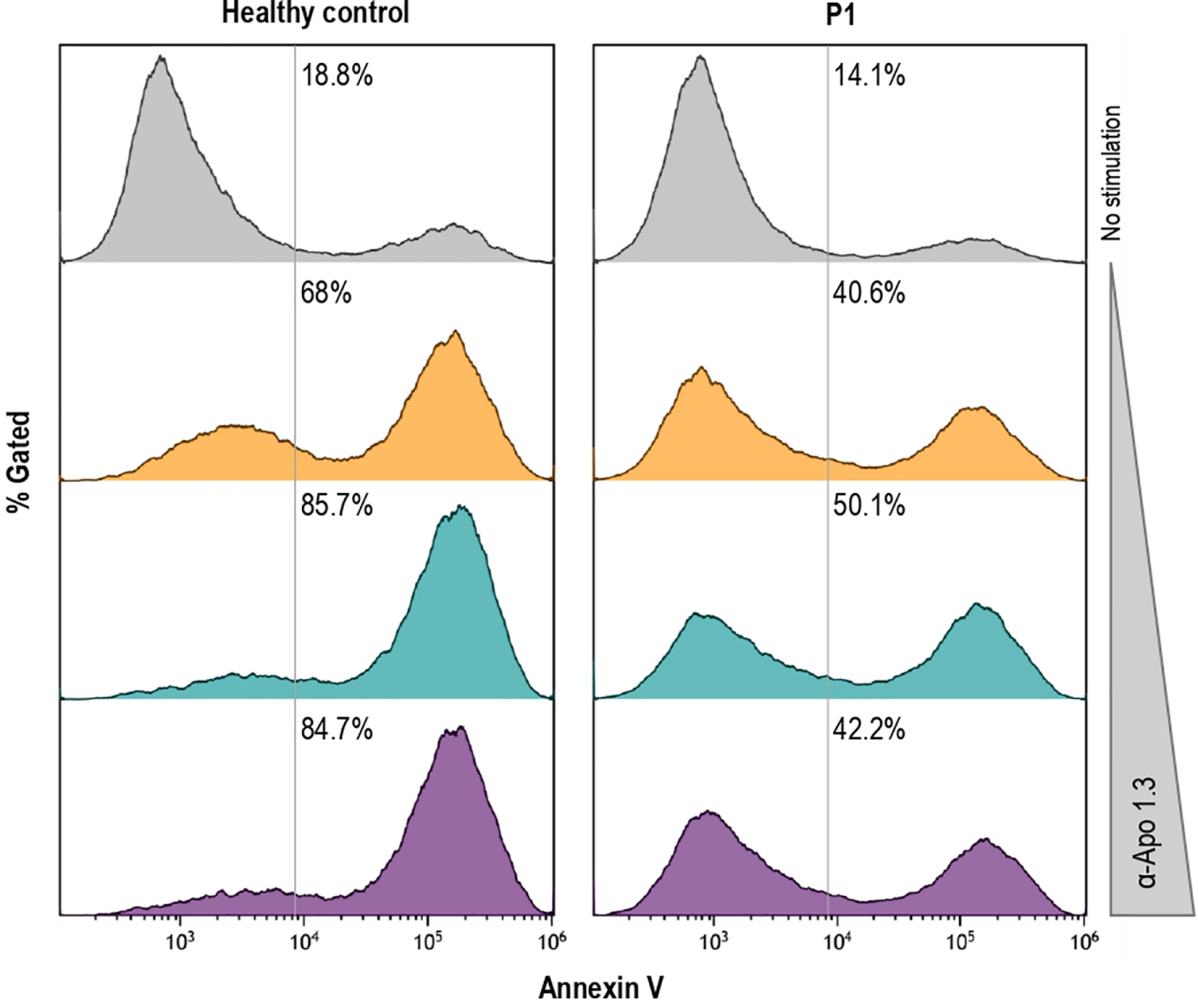
Induction of FAS-mediated apoptosis by different doses of agonistic anti-FAS mAb in PHA-activated T-cell blasts, as previously described in other ALPS patients (14), from the healthy control and P1 is shown.

Apoptosis testing is an intensive, costly labor, available only in a few specialized centers and not really helpful in routine evaluation of patients. According to the proposed ALPS diagnostic algorithms, the presence of a pathogenic mutation in ALPS-related genes establishes a diagnosis of ALPS. Although in some circumstances, FAS-mediated apoptosis assay can be useful to clarify the pathogenicity of variants of uncertain significance (VUS). Interestingly, FAS-mediated apoptosis induced with anti-FAS mAb in T-cell blasts was partially reduced in P1 (**Figure 3**) as previously described in other ALPS-sFAS patients (13).

## Discussion

ALPS is mostly caused by *FAS* mutations either germline or somatic, being restricted mainly to DNT in the latter case. Due to both ALPS-FAS and ALPS-sFAS patients cannot be clinically differentiated, sequencing of *FAS* gene in DNA from isolated DNT is recommended for ALPS-suspected patients without molecular diagnosis (30). However, the time, cost and technical requirements makes the DNT sorting a technique not accessible to all laboratories.

NGS has been demonstrated to be a powerful tool that enables to detect very low allele frequency variants. Detecting somatic mutations by NGS is a critical part of cancer research and diagnostics (31–33). Sequencing depth is required since mutations need to be observed on a sufficient number of reads to pass predetermined variant calling threshold (21). Thus, targeted gene panel sequencing is the optimal option to allow very deep coverage in a cost-effective way. Increasingly, new approaches are being developed for the exploration of somatic disease-causing mutations, being more frequent than expected. High-throughput NGS is presented as an important tool for the discovery of new diseases caused by postzygotic mosaicism (34–38). Hence, there is a role for customized targeted NGS approach in some circumstances looking for somatic variants when germline variant calls remain noncontributory and might help correlate clinical and cellular phenotypes with genomic datasets.

In an attempt to facilitate the molecular diagnosis of ALPS-sFAS patients, we describe clinical, immunologic and molecular findings of a new ALPS-sFAS patient diagnosed through NGS targeted gene panel. The somatic c.651+2T>C mutation affecting the intracellular domain of the *FAS* gene was detected with a somatic variant caller plugin as well as six other ALPS-sFAS previously diagnosed (13, 24). The mutation identified in this domain has the highest clinical penetrance (>80%) because it exerts dominant-negative effect on wild-type FAS protein (14). This strategy can be very useful allowing a first screening of possible somatic variants for their subsequent confirmation by Sanger sequencing in isolated DNT. This approximation could guide the next diagnostic steps and help assess whether DNT isolation is necessary, especially in the case of pediatric patients, as P1, where the limitation of the significant amount of blood needed, together with the technical requirements for the isolation of DNT, can lead in many cases to patient mismanagement (39).

As limitations of this study, we could consider that in the case of T-cell lymphopenia of the ALPS patients, the detection of the somatic FAS mutation would be more difficult in the whole blood (P1-P8 did not show T-lymphopenia). This fact must be considered in the case of ALPS patients under treatment when the diagnosis has been delayed for several circumstances. By the contrary, at diagnosis ALPS patients show normal/high numbers of T-lymphocytes.

Other limitation of the study is in case of ALPS patients with DNT percentages in the limit of the normal values (**Table 1**). In the case of P8, an ALPS-sFAS patients with borderline DNT (**Table 1**) and presenting a complete codon deletion (c.812_814del; p.Ala271del) (25), the mutation was not detected with the variant caller plugin used. However, the mutations of P1 and P4, also presented with borderline DNT (**Table 1**), were correctly detected. Probably a combination of the lowest DNT percentage and the nature of the P8 mutation are the main limitations for detecting the mutation by NGS.

Somatic mutation in *FAS* or in combination with germline mutation in genes other than *FAS* as *XIAP, PRF1, UNC13D* or *CASP10* have been reported (11–13, 15, 40). The second mutation is not an ALPS-causative hit and the codified proteins are not involved directly in FAS-FASL pathway but have been proposed as ALPS-phenotype modifiers (11, 12, 15).

In this work, in addition to the somatic *FAS* mutation causing ALPS-sFAS detected in P1, a germline variant caller plugin revealed two heterozygous mutations in *PRF1* and *UNC13D* genes predicted as possibly pathogenic, but not sufficient to cause hemophagocytic lymphohistiocytosis (41, 42) due to the autosomal recessive form of the disease. Furthermore, the expression of perforin and degranulation in NK cells were preserved in the patient, and probably these variants at best contribute marginally to clinical phenotype.

In summary, we report the first ALPS patient with a somatic *FAS* mutation detected through NGS targeted gene panel. Identification of somatic *FAS* mutations with this novel strategy, even in the cases of borderline DNT, is a cost and time-effective option to expedite the diagnosis and treatment of these patients avoiding further complications due to a delayed diagnosis.

## Data Availability Statement

The datasets presented in this study can be found in online repositories. The name of the repository and accession numbers can be found below: National Center for Biotechnology Information (NCBI) GenBank, https://www.ncbi.nlm.nih.gov/genbank/, MW674786, MW674787, MW674788 and MW674789.

## Ethics Statement

The studies involving human participants were reviewed and approved by Comité Ético de Investigación Clínica, University Hospital 12 de Octubre and by ethic committee of CPP Ile-de-France (CPP IDF2 DC-2014-22722015-03-03 AF). Written informed consent to participate in this study was provided by the participants’ legal guardian/next of kin.

## Author Contributions

ML-N designed the pipeline, analyzed the results and drafted the manuscript, tables, and figures. JD-C designed the bioinformatics algorithm. JR, RR-P, CG-L, SS-R, JGH, AM and FR-L conducted the clinical and immunological follow-up of the patient. MJD-M, MAD-M, PMP, EP-A, AM and FR-L did the molecular (NGS and Sanger sequencing) and functional studies (immunophenotyping, biomarkers and functional studies of apoptosis). LA conceived the idea for the manuscript and drafted the manuscript, tables, and figures. All authors contributed to the article and approved the submitted version.

## Funding

This work was supported by grants from Fondo de Investigación Sanitaria (FIS-PI16/2053) to LA. The project has been co-financed with FEDER funds. ML-N was co-financed by “Fondo Social Europeo, Programa Operativo de empleo juvenil (YEI)”. The study was also supported by the Institut National de la Santé et de la Recherche Médicale (INSERM) and by government grants managed by the Agence National de la Recherche as part of the “Investment for the Future” program (ANR-10-IAHU-01 and ANR-18-RHUS-0010), the Ligue Contre le Cancer—Comité de Paris, Fondation ARC pour la recherche sur le CANCER, the Centre de Référence Déficits Immunitaires Héréditaires (CEREDIH), the Agence National de la Recherche (ANR-14-CE14-0026-01 “Lumugene”; ANR-18-CE17-0001 “Action”).

## Conflict of Interest

The authors declare that the research was conducted in the absence of any commercial or financial relationships that could be construed as a potential conflict of interest.

## Abbreviations

ALPS, Autoimmune Lymphoproliferative Syndrome; DNT, Double Negative T-cells; ELISA, Enzyme-Linked Immunosorbent Assay; FADD, FAS-Associated protein with Death Domain; FASL, FAS Ligand; Ig, Immunoglobulin; IGV, Integrative Genomics Viewer; IL-10, Interleukin-10; IUIS, International Union of Immunological Societies; MACS, Magnetic-Activated Cell Sorting; NGS, Next Generation Sequencing; PBMC, Peripheral Blood Mononuclear Cells; PID, Primary Immunodeficiency; PIRD, Primary Immune Regulatory Disorder; sFASL, Soluble FAS Ligand; TMAP, Torrent Mapping Alignment Program; VAF, Variant Allele Frequency; VUS, Variant of Uncertain Significance.
